# Modelling the dynamics of *Plasmodium falciparum *histidine-rich protein 2 in human malaria to better understand malaria rapid diagnostic test performance

**DOI:** 10.1186/1475-2875-11-74

**Published:** 2012-03-19

**Authors:** Louise Marquart, Alice Butterworth, James S McCarthy, Michelle L Gatton

**Affiliations:** 1 Statistics Unit, Queensland Institute of Medical Research, Herston, Australia; 2 Clinical Tropical Medicine Laboratory, Queensland Institute of Medical Research, Herston, Australia; 3 School of Medicine, University of Queensland, Herston, Australia; 4 Malaria Drug Resistance and Chemotherapy Laboratory, Queensland Institute of Medical Research, Locked Bag 2000, Royal Brisbane Hospital, HerstonQld 4029, Australia

**Keywords:** Histidine-rich protein, Rapid diagnostic tests, *Plasmodium falciparum*

## Abstract

**Background:**

Effective diagnosis of malaria is a major component of case management. Rapid diagnostic tests (RDTs) based on *Plasmodium falciparum*histidine-rich protein 2 (*Pf*HRP2) are popular for diagnosis of this most virulent malaria infection. However, concerns have been raised about the longevity of the *Pf*HRP2 antigenaemia following curative treatment in endemic regions.

**Methods:**

A model of *Pf*HRP2 production and decay was developed to mimic the kinetics of *Pf*HRP2 antigenaemia during infections. Data from two human infection studies was used to fit the model, and to investigate *Pf*HRP2 kinetics. Four malaria RDTs were assessed in the laboratory to determine the minimum detectable concentration of *Pf*HRP2.

**Results:**

Fitting of the *Pf*HRP2 dynamics model indicated that in malaria naïve hosts, *P. falciparum *parasites of the 3D7 strain produce 1.4 × 10^-13 ^g of *Pf*HRP2 per parasite per replication cycle. The four RDTs had minimum detection thresholds between 6.9 and 27.8 ng/mL. Combining these detection thresholds with the kinetics of *Pf*HRP2, it is predicted that as few as 8 parasites/μL may be required to maintain a positive RDT in a chronic infection.

**Conclusions:**

The results of the model indicate that good quality *Pf*HRP2-based RDTs should be able to detect parasites on the first day of symptoms, and that the persistence of the antigen will cause the tests to remain positive for at least seven days after treatment. The duration of a positive test result following curative treatment is dependent on the duration and density of parasitaemia prior to treatment and the presence and affinity of anti-*Pf*HRP2 antibodies.

## Background

*Plasmodium falciparum *causes the most severe form of malaria, and contributes to high morbidity and mortality in areas of the world where malaria is endemic. Accurate diagnostics are paramount for efficient treatment of falciparum malaria. In this context, the popularity of malaria rapid diagnostic tests (RDTs) has increased due to the WHO recommendation for parasitological confirmation of suspected malaria cases prior to treatment [1]. Compared to microscopy, RDTs are easier to use, do not require well-maintained microscopes and skilled microscopists, and provide a timely result. The majority of *P. falciparum *specific RDTs function by detecting a water soluble protein produced by *P. falciparum*, namely histidine-rich protein 2 (*Pf*HRP2). Detection is accomplished using monoclonal antibodies specific for *Pf*HRP2. *Pf*HRP2 is produced by the parasite throughout its asexual lifecycle; it is expressed on the surface of infected erythrocytes and released into the peripheral circulation during schizogony [2,3]. Due to the persistence of the protein in the circulation, *Pf*HRP2-detecting RDTs have however been reported to have low specificity for diagnosis of current malaria infection in areas of high transmission [4] and following treatment [5,6]; that is, the *Pf*HRP2-detecting RDTs may return positive results when parasites are not present in the blood. This persistence also affects the suitability of *Pf*HRP2-detecting RDTs for diagnosis of malaria in endemic regions where chronic asymptomatic infections are common. In addition, the timing of onset of detectable antigenaemia using the RDT, relative to the appearance of parasites in the bloodstream at a level detectable by standard microscopic examination of a thick blood film is not well defined.

As mature *P. falciparum *parasites sequester in splanchnic vascular beds during the last half of the asexual life-cycle they are not accessible for microscopic diagnosis at this time. An additional consequence of sequestration is that the total parasite biomass may be underestimated if peripheral blood sampling is relied upon. It has, therefore, been proposed that quantitative assays of *Pf*HRP2 level provide a more accurate measurement of parasite biomass and potentially assist in determining the prognosis of severe malaria [2,7].

The kinetics of *Pf*HRP2 antigenaemia are not well understood, although key parameters such as rate of production and elimination half-life have been estimated from in vitro and in vivo studies [7,8]. In the current study a model of the kinetics of *Pf*HRP2 is developed and used to assess its potential impact on the interpretation of malaria RDT results. This model was applied to clinical data to estimate circulating *Pf*HRP2 levels, as well as the amount of *Pf*HRP2 available for detection using RDTs that include a red blood cell (RBC) lysis step.

## Methods

### Modelling PfHRP2 kinetics

A mathematical model was developed to mimic the kinetics of *Pf*HRP2, incorporating both the production and clearance of the protein during a *P. falciparum *infection. In developing the model it was assumed that the host was infected with a single, relatively synchronous infection of *P. falciparum *with a two-day (48 hour) asexual life cycle.

Studies of in vitro culture of *P. falciparum *indicate that *Pf*HRP2 accumulates in the cell as the parasite develops within the erythrocyte with little membrane leakage, resulting in 78-99% of *Pf*HRP2 being released into the circulation when the erythrocyte ruptures during schizogony [2]. The model assumed production of *PfHPR2 *occurred early in the asexual cycle, with all the protein remaining within the infected RBC (iRBC) until the time of schizont rupture when it is instantaneously released into the circulation. The number of parasites replicating (i.e. schizonts rupturing) at a given time during an infection was estimated from available clinical data. Where required, parasite density was converted to absolute number of parasites by multiplying the density by an assumed blood volume of 5 L (5 × 10^6 ^μL).

Two scenarios for *Pf*HRP2 distribution within the body were considered; 1) the minimum circulating concentration, estimated assuming that *Pf*HRP2 is water-soluble and distributed in equilibrium throughout the extracellular water volume of 14 L [9], 2) maximum circulating concentration, estimated assuming *Pf*HPR2 is distributed only within the total blood volume of 5 L.

The elimination of *Pf*HRP2 from the circulation was assumed to be continuous following a first-order decay process:

(1) $$d_{k}=\text{exp}\left[\frac{\text{ln}\left(0.5\right)k}{3.67}\right]=\text{exp}\left[-0.18887k\right]$$

where *d_k _*is the proportion of *Pf*HRP2 remaining *k *days after it was produced (*k *= 0,..., 25). The exponent was based on the published *Pf*HRP2 half-life of 3.67 days [7].

The absolute amount of *Pf*HRP2 (in grams) for any day during an infection (*H_t_*) was calculated using equation (2), while the minimum (*Hmin_t_*) and maximum (*Hmax_t_*) circulating concentrations of *Pf*HRP2 (g/μL) were calculated according to equations (3) and (4), respectively.

(2) $$H_{t}=f\overset{t}{\underset{j=1}{\sum }}d_{t-j}p_{j}$$

where *t *is the time in days with *t *= 1 being the first schizogony of the parasite, *p_j _*is the number of parasites within the host replicating on day *j*, and *f *is the amount of *Pf*HRP2 produced per iRBC (g).

(3) $$Hmin_{t}=\frac{H_{t}}{14\times 10^{6}}$$

(4) $$Hmax_{t}=\frac{H_{t}}{5\times 10^{6}}$$

The concentration of *Pf*HRP2 measured by RDT or ELISA (*Tmin_t _*and *Tmax_t_*) exceeds *Hmin_t _*and *Hmax_t _*since it contains the circulating *Pf*HRP2 plus the *Pf*HRP2 within the iRBCs which is released due to cell lysis as a consequence of an RDT or ELISA assay.

(5) $$Tmin_{t}=Hmin_{t}+\frac{fq_{t}}{5\times 10^{6}}$$

(6) $$Tmax_{t}=Hmax_{t}+\frac{fq_{t}}{5\times 10^{6}}$$

where *q_t _*is the number of parasites within the host on day *t *(*q_t _*= *p_t _*on replication days in a synchronous infection).

### Study datasets

To fit the model and assess sensitivity, the *Pf*HRP2 kinetics model was applied to two datasets.

Study 1. The first dataset was from a prospective, unblinded, Phase IIa clinical trial where volunteers were infected with blood stage *P. falciparum *[10]. The subjects were infected with approximately 1,800 *P. falciparum *(3D7 strain) asexual parasites, and were given curative treatment soon after reaching the target parasitaemia of ≥ 1,000 parasites/mL, as determined by PCR quantification [11]. Details of the study, including ethics approval have been previously reported [10]. For the current study data from subjects in the third study cohort were considered, and of the nine subjects in this cohort, three (subjects 12, 13 and 14) had sufficient numbers of pre-treatment data points to calibrate the model. Parasite density and corresponding *Pf*HRP2 concentration were estimated from blood samples taken at pre-specified time points (approximately once or twice daily) from initial infection to parasite clearance.

To determine the concentration of circulating *Pf*HRP2, serial blood samples from each subject were assessed by *Pf*HRP2 ELISA (Malaria Ag. Pf. ELISA Standard Diagnostics Korea; product code 05EK50). To interpolate the amount of *Pf*HRP2 present in the blood samples, a calibration curve was constructed using serial dilutions of a 3D7 *P. falciparum*culture supernatant and used as control standards in each ELISA. The concentration of *Pf*HRP2 in this culture supernatant had previously been measured at 55.5 ng/mL by interpolating the ELISA optical density of serial dilutions against a stock of recombinant *Pf*HRP2 protein with known concentration (Lee, personal communication). *Pf*HRP2 concentration within each sample was determined by interpolating the optical density of the sample with the standard curve using the software package Softmax Pro (Molecular Devices Inc.).

The pre-treatment parasitaemia data was used to fit the *f *parameter in the maximum concentration model. As the *Pf*HRP2 quantification was conducted using RBC pellets, rather than the serum, only the maximum concentration model was considered during the model fitting. An initial value of *f *(*f_0_*) was assumed to be 5.2 × 10^-15 ^g, as this was the median value previously reported for four *P. falciparum *isolates during in vitro culture [8]. Calibration of the model to calculate the optimal value for *f *(f*) was achieved by determining the multiplication factor (*m*) which produced the minimum residual sum of squares between the predicted *Pf*HRP2 concentration and subject ELISA data such that f* = f_0_m.

Study 2. The second dataset was a sample of three patients with neurosyphilis who had been treated by infection with *P. falciparum *[12]. Patients S561, S707 and S811 were selected as illustrative examples of a 'natural infection', as the malaria infection was not treated with antimalarial drugs to modify the primary attack. Patients S561 and S811 had no previous history of malaria infection and were infected with the McLendon strain of *P. falciparum *(blood-induced), whilst Patient S707 was reinfected with the McLendon strain of *P. falciparum *after being infected with the same strain 30 days prior [13]. The parasitaemia data from this study set was used to investigate the kinetics of *Pf*HRP2 antigenaemia in symptomatic, untreated infections.

### Threshold of detection of malaria RDTs

To assess the minimum *Pf*HRP2 concentration detectable by malaria RDTs four different RDT products were tested against serial dilutions of 3D7 parasite culture supernatant containing a known concentration of *Pf*HRP2. This supernatant was the same used to generate the standard curve in the ELISA assay described for Study 1 above. The products tested were Firstsign™ - ParaView (Pan + Pf) Malaria Test (Catalogue # 2101 CB-25, Unimed International Inc), SD BIOLINE Malaria Ag Pf/Pv (Catalogue # 05FK80, Standard Diagnostics Inc), Carestart™ Malaria HRP2/pLDH (Pf/PAN) COMBO (Catalogue # G0131, AccessBioInc) and ICT Malaria Combo Cassette Test (Catalogue #ML02, ICT Diagnostics).

Each sample with a specified concentration of *Pf*HRP2 was added to the RDTs as per the manufacturer's instructions with the test scored as positive if the *Pf*HRP2 band was visible and negative if there was no visible *Pf*HRP2 band. The reader of the RDT was not blinded to the concentration of *Pf*HRP2 added to the RDT. The maximum dilution producing a positive result was classified as the RDT detection threshold, and the concentration of *Pf*HRP2 corresponding to this dilution was calculated.

## Results

### Estimation of amount of PfHRP2 produced by parasites

**Figure 1:**
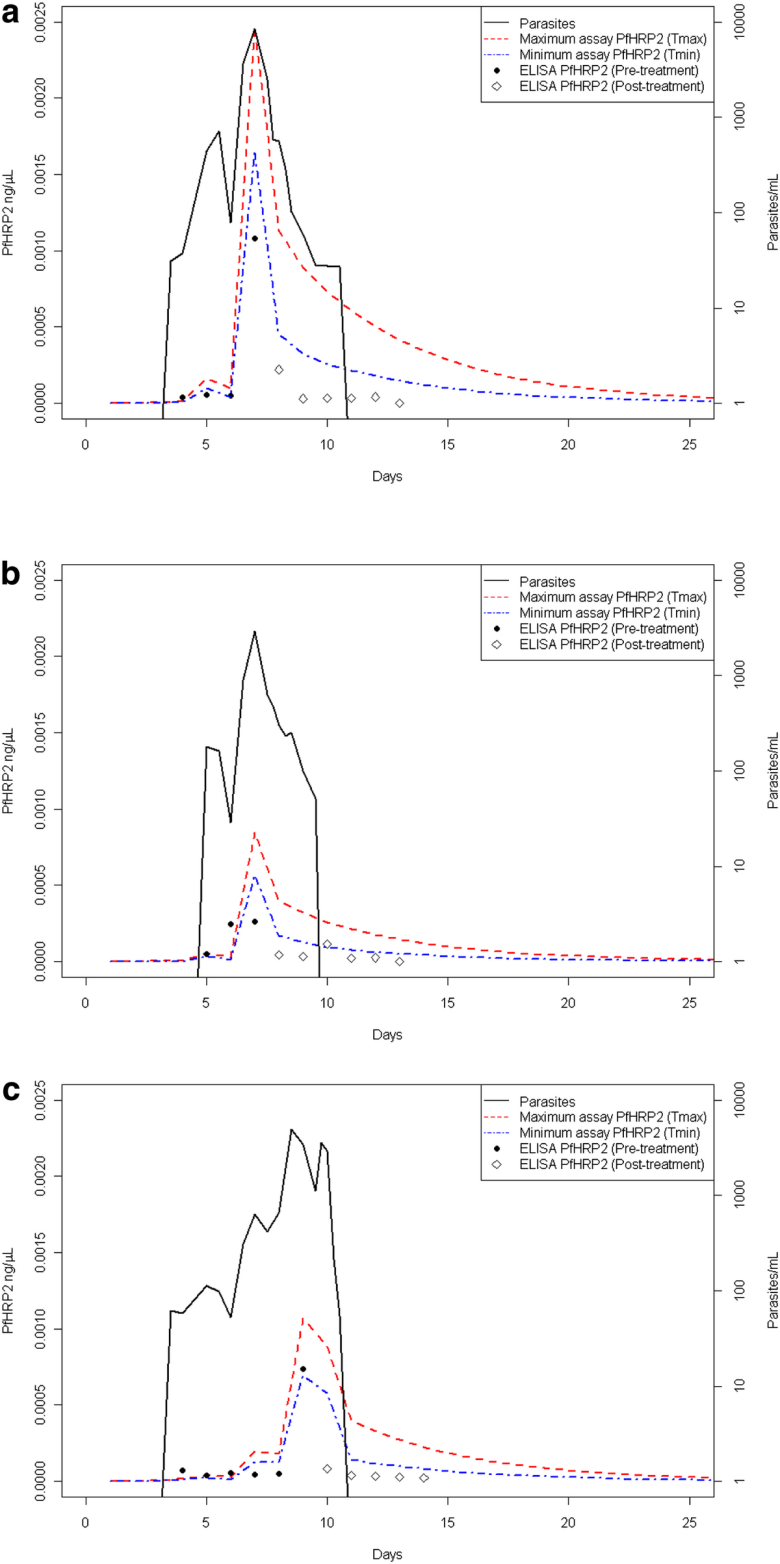
**Parasitaemia and ELISA data from Study 1, along with the predicted*Pf*HRP2 concentration**. Data are presented for a) Patient 12, b) Patient 13 and c) Patient 14. The *Pf*HRP2 concentrations were predicted by applying the fitted model with the mean optimal *Pf*HRP2 production of 1.4 × 10^-13 ^g per parasite per replication cycle.

The *Pf*HRP2 model was calibrated using data from Study 1. The optimal individualized multiplication factor (*m*) for subjects varied between 17.5 and 39.4, suggesting that the amount of *Pf*HRP2 produced by the 3D7 *P. falciparum *parasites was considerably higher than previously reported (Table 1). The mean multiplication factor for the three subjects was 27.1, resulting in an estimate of 1.4 × 10^-13 ^g of *Pf*HRP2 produced per parasite per replication cycle. The fit of the predicted *Pf*HRP2 quantities for each individual using the mean optimal multiplication factor is presented in Figure 1.

**Table 1:** Optimal multiplication factor for production of *Pf*HRP2 by 3D7 *P.falciparum *parasites

Subject	Treatment day	No. pre-treatment data points	Optimal multiplication factor (*m*)	Estimated *Pf*HRP2 produced/parasite (f*)
12	7.5	4	24.4	127 fg

13	7.5	3	17.5	91 fg

14	9.5	6	39.4	205 fg

The estimated quantity of *Pf*HRP2 produced per parasite is calculated as *m *x 5.2 fg.

### Kinetics of PfHRP2 in untreated infections

**Figure 2:**
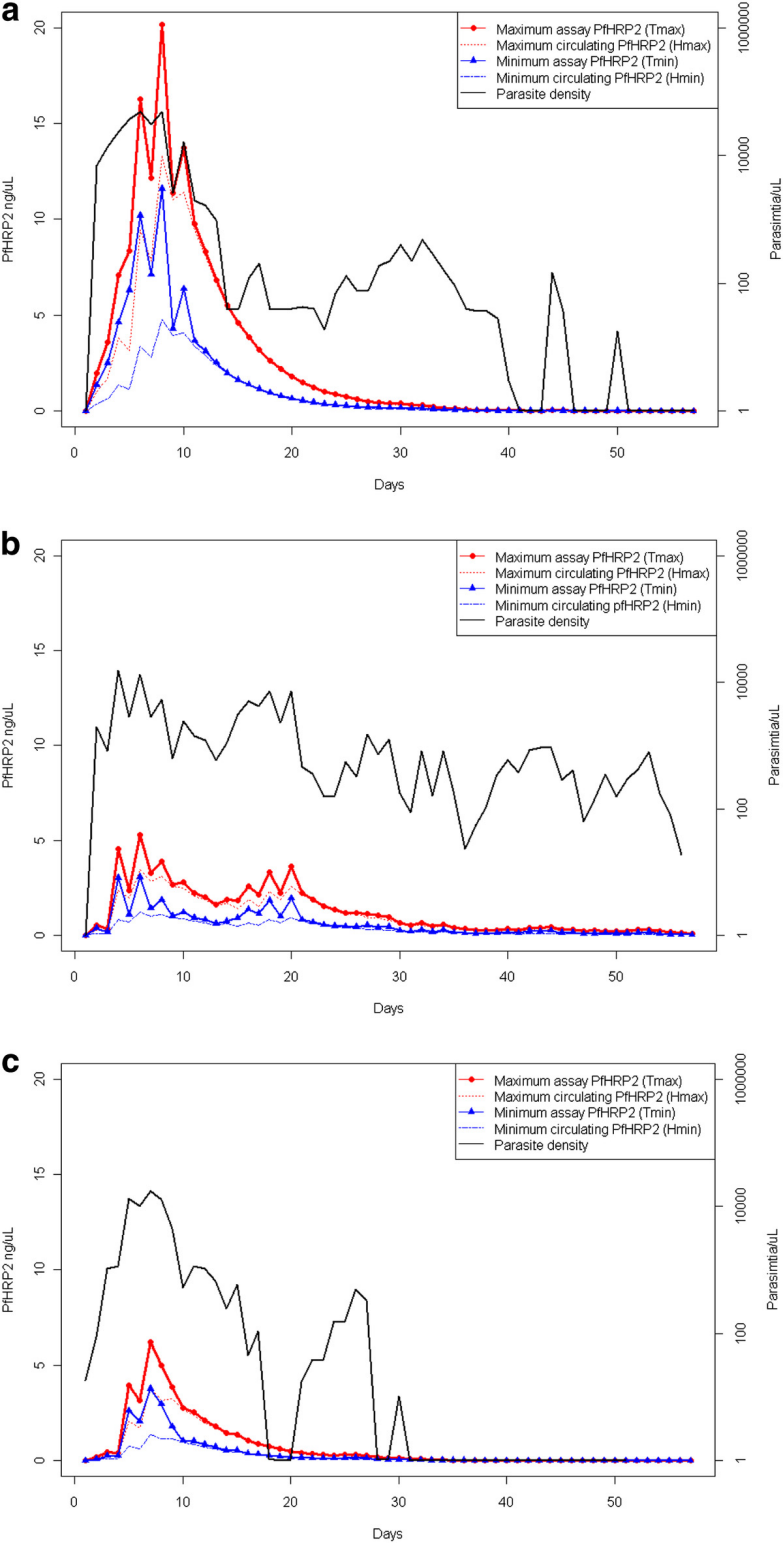
**Parasitaemia and estimated*Pf*HRP2 concentrations over the course of untreated infections**. Data are presented for patients a) S561, b) S707 and c) S811 in Study 2. Predicted *Pf*HRP2 concentrations are for the maximum (*H_max_*) and minimum (*H_min_*) circulating *Pf*HRP2, and the *Pf*HRP2 concentration which would be measured during and ELISA or RDT (*T_max _*and *T_min_*) which includes intracellular *Pf*HRP2 which is released during the assay.

The model was used to investigate the kinetics of *Pf*HRP2 antigenaemia during an infection by using parasitaemia data from the three neurosyphilis patients (S561, S707 and S811) in Study 2. The amount of circulating *Pf*HRP2 (*Hmin_t _*and *Hmax_t_*), as well as the amount of *Pf*HRP2 predicted to be measurable by ELISA (*Tmin_t _*and *Tmax_t_*) were calculated (Figure 2).

The duration and first day that *Pf*HRP2 could be detected at concentrations between 10 ng/mL and 100 ng/mL were estimated for each patient. Due to the potential for different *P. falciparum *parasite strains to produce different quantities of *Pf*HRP2 (*f*), the model was applied using the optimal value determined in Study 1 (fitted model, *f *= 1.4 × 10^-13 ^g) as well as the previously reported value of 5.2 × 10^-15 ^g [8] (unadjusted model) (Table 2). Considering a threshold of 100 ng/mL, the first day that *Pf*HRP2 was predicted to be detectable was Day 2 for all three patients under the fitted model, and Day 4 or Day 7 under the unadjusted model for patients S561 and S811, and S707, respectively. For the fitted model, the number of days that *Pf*HRP2 was predicted to be greater than 100 ng/mL ranged between 25 and 50 days under the minimum circulation model and between 29 and 56 days under the maximum circulation model. The duration during an infection when the *Pf*HRP2 antigenaemia was above the nominated thresholds was always longer for the maximum circulation model compared to the minimum circulation model, and the model output was highly sensitive to changes in *f*. The fitted model always produced a longer period of antigenaemia above a threshold, and an equal or earlier transition to this state, than the unadjusted model, due to the higher production of *Pf*HRP2 per parasite per replication cycle.

**Table 2:** Characteristics of predicted *Pf*HRP2 kinetics in illustrative untreated infections

Patient	Day of first fever	*Pf*HRP2 threshold (ng/mL)	Fitted model		Unadjusted model	
			
			First day above threshold (MinCM, MaxCM)	Days above threshold (range)	First day above threshold (MinCM, MaxCM)	Days above threshold (range)
S561	2	100	2,2	32-34	4,3	9-15

		20	2,2	39-46	2,2	19-25

		10	2,2	44-51	2,2	24-31

S707	4	100	2,2	25-29	7,5	2-6

		20	2,2	31-35	5,5	10-15

		10	2,1	34-40	5,3	13-25

S811	2	100	2,2	50-56	4,4	2-7

		20	2,2	59-64	4,2	21-31

		10	2,2	63-68	2,2	29-47

The kinetics are summarized by the day they first exceed a nominated threshold under the minimum (MinCM) and maximum circulating *Pf*HRP2 models (MaxCM) and the total number of days they are above this threshold. A range for the number of days above the threshold is given by combining the results from the MinCM and MaxCM models. Results are presented using the mean *Pf*HRP2 production fitted in this study (fitted model) and also the previously reported *Pf*HRP2 production of 5.2 fg/parasite/replication cycle (unadjusted model) [8].

The parasitaemia time-course from subjects in Study 1 was used to assess the longevity of circulating P*f*HRP2 following curative treatment. In these three subjects the amount of *Pf*HRP2 decayed to be less than 110 ng/L 12 days, 7 days and 8 days after curative treatment for subjects 12, 13 and 14, respectively.

### Chronic infections - the minimum number of parasites required to maintain PfHRP2 above a threshold

**Figure 3:**
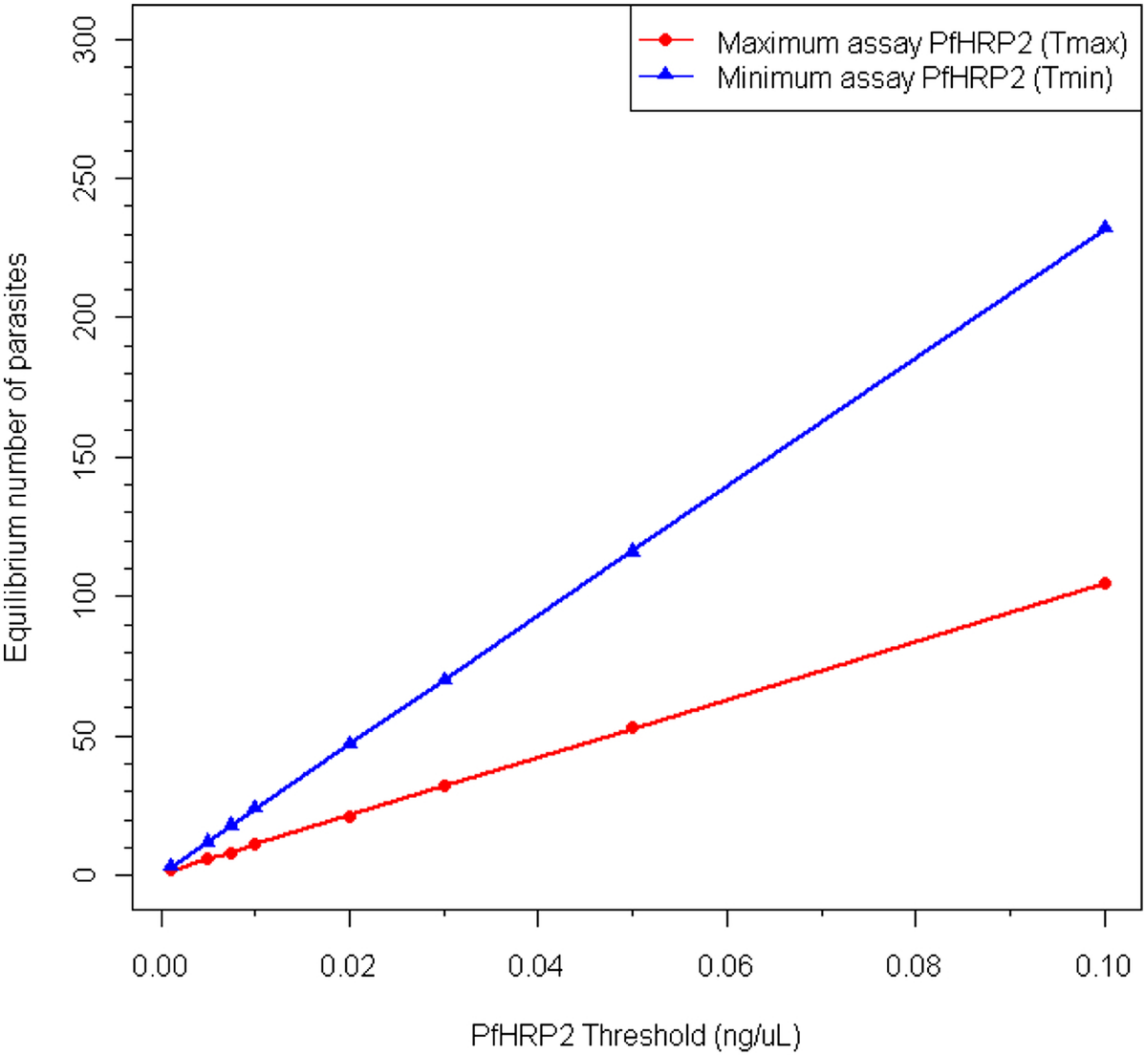
**Predicted minimum number of*P. falciparum*parasites required to maintain*Pf*HRP2 concentration above a specified threshold**. Data are presented for the fitted maximum circulating model (*T_max_*, red) and minimum circulating model (*T_min_*, blue).

The equilibrium parasite density required to maintain the *Pf*HRP2 level above a nominated threshold was also determined for both the minimum and maximum circulation models (Figure 3). Applying these parasite densities resulted in the concentration of *Pf*HRP2 gradually increasing until approximately Day 20, after which it plateaus at the equilibrium value. Linear regression models were fitted to determine the relationship between the threshold concentration (ng/μL) and equilibrium parasite density (parasites/μL). Statistically significant models were achieved (P < 0.001) and are reported below. The values in brackets represent the SE of the coefficients.

Equilbrium no. of parasites(minimum model)= 0.7+2311.9×PfHRP2 threshold(0.1)   (2.3)

Equilbrium no. of parasites(minimum model)= 0.6+1044.3×PfHRP2 threshold(0.1)   (3.4)

These estimates of the equilibrium number of parasites represent the minimum number of parasites required consistently over time to maintain the level of *Pf*HRP2 above a nominated threshold. Parasite densities above the equilibrium would always produce *Pf*HRP2 levels over the threshold. Fluctuating parasite densities, as may be seen in chronic infections, will also maintain *Pf*HRP2 levels above the threshold provided the period of parasitaemia below the equilibrium value is short; the maximum duration of low parasitaemia will be dependent on the parasite dynamics and amount of *Pf*HRP2 produced immediately prior to the drop in parasite density.

### Malaria RDT detection thresholds

The performances of each RDT against known *Pf*HRP2 concentrations are shown in Table 3. The lowest dilutions at which positive test results occurred were 27.75 ng/mL, 6.94 ng/mL and 6.94 ng/mL for the Firstsign™ - ParaView (Pan + Pf) Malaria Test, SD BIOLINE Malaria Ag Pf/Pv, and Carestart™ Malaria HRP2/pLDH (Pf/PAN) COMBO (Catalogue # G0131, AccessBioInc), respectively. The ICT Malaria Combo Cassette Test produced a faint positive band when tested against the negative control (culture media) and the lowest *Pf*HRP2 dilutions (Table 3). The band intensity for this product increased when tested with a sample containing 13.88 ng/mL of *Pf*HRP2, so this concentration was considered to be the minimum level of detection for this product.

**Table 3:** Malaria RDT reactivity against*P.falciparum*culture supernatant

Product	Intensity of *Pf*HRP2 band						
	
	27.75 ng/mL	13.88 ng/mL	6.94 ng/mL	3.47 ng/mL	1.73 ng/mL	0.87 ng/mL	0 ng/mL (-ve control)
Firstsign™ - ParaView (Pan + Pf) Malaria Test	1	Neg	Neg	Neg	Neg	Neg	Neg

SD BIOLINE Malaria Ag Pf/Pv	4	2	1	Neg	Neg	Neg	Neg

Carestart™ Malaria HRP2/pLDH	1	1	1	Neg	Neg	Neg	Neg

ICT Malaria Combo Cassette Test	4	2	1	1	1	1	1

Table entries are the intensities of the *Pf*HRP2 band, scored from 1 (faint band) to 4 (strong band). Neg indicates no *Pf*HRP2 band was visible.

Applying these detection thresholds to the estimated equilibrium equations above indicates that the threshold parasitaemia that would be required to maintain a positive RDT in a chronic infection would be between 8 and 65 parasites/μL, dependent on the distribution of *Pf*HRP2 within the body and the specific product (Table 4).

**Table 4:** Predicted equilibrium parasite densities required to maintain a positive RDT test

Product	Min. detectable *Pf*HRP2 conc. (ng/mL)	Est. no. parasites for MinCM (parasites/μL)	Est. no. parasites for MaxCM (parasites/μL)
Firstsign™ - ParaView (Pan + Pf) Malaria Test	27.75	64.9	29.6

SD BIOLINE Malaria Ag Pf/Pv	6.94	16.7	7.8

Carestart™ Malaria HRP2/pLDH	6.94	16.7	7.8

ICT Malaria Combo Cassette Test	13.88*	32.8	15.1

* The lowest dilution which gives a band intensity > 1 to adjust for false positive result on negative control.

MinCM and MaxCM represent the minimum circulating model and maximum circulating model, respectively.

## Discussion

Understanding the kinetics of *Pf*HRP2 is important to understanding the performance characteristics of malaria RDTs that detect this antigen. For optimal clinical diagnosis RDTs need to return a positive result at the time of first fever in malaria naïve individuals. In contrast, the slow decay of the antigen has implications for the utility of *Pf*HRP2-based RDTs in endemic countries where low level infections are prevalent, and for the detection of recrudescent infections following treatment [5,6].

A key parameter in modelling the kinetics of *Pf*HRP2 antigenaemia is the amount of *Pf*HRP2 produced by one parasite during one replication cycle (*f*). It has been previously reported that each *P. falciparum *parasite produces 5.2 fg (range, 1.1 - 13.0 fg) of *Pf*HRP2 per cycle [8]. However, when this production level was used in the model, the concentration of *Pf*HRP2 was predicted to be considerably lower than that actually measured in malaria naïve individuals infected with blood-stage 3D7 *P. falciparum *parasites. This difference may be a consequence of the differences between testing antigen production from cultures grown in vitro compared to in vivo measurements, a consequence of measurement error and stochastic variability, or the result of natural variation in protein production between different parasite strains [14]. The subjects in Study 1 were all infected by the same 3D7 parasite stain so genetic variability in the production of *Pf*HRP2 should be minimal. Therefore, the two-fold variation between the calculated optimal values for the amount of *Pf*HRP2 produced per parasite per replication cycle is likely a result of measurement error in the low parasite densities or ELISA assay, or stochastic variability in *Pf*HRP2 production by individual parasites, possibly in response to host factors.

The model developed was applied under two assumptions: 1) a minimum concentration model in which the *Pf*HRP2 was assumed to be distributed throughout the extracellular water of the host and 2) a maximum concentration model in which the *Pf*HRP2 was assumed to be restricted to the bloodstream only. It is highly likely that the actual distribution of *Pf*HRP2 is somewhere between these two extremes so the values presented should be viewed in this context. For simplicity, it was assumed in the model that *Pf*HRP2 was produced early in the asexual life cycle, although in vitro data indicate that *Pf*HRP2 accumulates throughout the cycle [2]. This assumption will not impact the calculations for the amount of circulating antigen, but may produce a slight overestimation of the amount of antigen measurable by RDT or ELISA. The magnitude of the overestimation will depend on the relative amount of antigen contained within the cells at the time of the assay compared to the amount of circulating antigen, factors dependent on the parasite density and duration of the infection prior to sampling.

It was recently reported that a commercial *Pf*HRP2 ELISA kit could detect *Pf*HRP2 concentrations down to 110 ng/L [15]. In the current study where four commercial RDTs were tested against the same *Pf*HRP2 antigen, the diagnostic sensitivity of the tests varied between 6.9 μg/L and 27.7 μg/L of *Pf*HRP2. The model output shows that for the fitted model, these values are achieved on, or immediately prior, to the first day of fever for the three patients in Study 2. In Study 1, the predicted amount of *Pf*HRP2 would have been insufficient to produce a positive RDT test, as subjects were treated before becoming symptomatic, with peak parasite densities less than 10 parasites/μL. Field data indicates that *Pf*HRP2-detecting RDTs can detect parasite densities less than 100 parasites/μL [16,17]. The model predictions indicate that the malaria RDTs tested here would be able to diagnose an infection at the time of first fever. A lower level of *Pf*HRP2 production as in the unadjusted model, or higher detection thresholds in the RDT may cause a delay in a positive RDT result.

The model informs understanding on how duration of infection and parasite density impact on the longevity of *Pf*HRP2 in natural infections. This replicates field data, where children with higher parasite densities at the time of treatment had a significantly longer antigen persistence compared to children with lower parasite densities [18]. In Study 1, subjects were treated prior to becoming symptomatic, and the peak parasitaemia was much lower than would occur in a natural infection. Thus, the longevity of *Pf*HRP2 in this circumstance is a likely underestimate of the expected duration of a detectable antigenaemia in symptomatic infection. Indeed, as few as eight parasites/μL may be required to maintain *Pf*HRP2 equilibrium above 6.9 μg/L using the parameter space described.

As has been reported before, the diagnostic sensitivity of malaria RDTs targeting *Pf*HRP2 is varied. The four products examined here have also been assessed as part of the WHO Malaria Rapid Diagnostic Test Programme where they were tested against wild-type isolates [19,20]. Each product performed well when tested against 200 *P. falciparum *parasites/μL, with overall positivity rates varying from 93.0% for Firstsign™ - ParaView (Pan + Pf) Malaria Test to 99.1% for Carestart™ Malaria HRP2/pLDH (Pf/PAN) COMBO. The results from this study are generally aligned with the product testing results, as the product with the lowest panel detection score, Firstsign™ - ParaView (Pan + Pf) Malaria Test, had the highest *Pf*HRP2 detection threshold. The modelling results indicate that these tests should continue to perform consistently well at concentrations in the order of 50-100 parasites/μL, or better in some cases. This result reflects a previous study in which the ICT Malaria Combo Cassette Test was able to detect *P. falciparum *parasites below 100/μL in field samples [17]. However it is important to note that the four products tested in the current study were within the top 40% of RDTs tested by the WHO programme, as ranked by their performance against wild-type *P. falciparum *isolates at 200 parasites/μL [21], and in addition were stored in ideal conditions. Therefore, extrapolation of the results obtained here to other RDTs is not recommended, particularly those products not achieving the same level of performance as the tested sub-set.

The model and results presented in the current study assume all *Pf*HRP2 antigen produced by parasites in natural human infection is available for measurement. However, it is possible that *Pf*HRP2 binds or complexes with other ligands or proteins such as *Pf*HRP2-specific antibodies, thereby reducing the amount available as free circulating antigen. In this respect, it is known that anti-*Pf*HRP2 antibodies develop and may thereby act to reduce the amount of free *Pf*HRP2 circulating [3]. While these factors have not been considered in the current model, it is likely their effect would primarily influence the longevity of the antigen in the circulation after treatment. As RDTs cause RBC lysis, while there is an active infection and parasites within the host, a blood sample tested using an RDT would be able to detect the *Pf*HRP2 contained within the iRBCs provided that the quantity of antibody in the serum is insufficient to bind all of the *Pf*HRP2 released from the iRBCs before it reacts with the test band in the RDT. A caveat to this is that the parasites must be present in the blood sample tested by the RDT. Parasite sequestration can affect the availability of parasites in finger-prick samples, a factor which has not been considered in this model.

## Conclusion

Malaria RDTs are becoming the cornerstone of effective malaria diagnosis in primary healthcare settings. Understanding the benefits and limitations of these tools is important to guide clinicians and product development. The outputs of the mathematical model reported here indicate that the *Pf*HRP2-based RDTs that were tested should be able to detect parasites on the first day of symptoms, and that the persistence of the antigen will cause the tests to remain positive for at least seven days post-treatment. The post-treatment duration of positive tests is dependent on the duration and density of parasitaemia prior to treatment, and possibility other factors such as anti-*Pf*HRP2 antibodies. In chronic infections a steady state parasitaemia of only 4-65 parasites/μL is likely to be sufficient to maintain a positive RDT result.

## Competing interests

The authors declare that they have no competing interests.

## Authors' contributions

LM developed the model, conducted the analysis and drafted the manuscript. AB performed the laboratory testing of RDTs and ELISA assays. JMC participated in the design of the study. MLG participated in the design of the study, reviewed the model output and helped draft the manuscript. All authors read and approved the final manuscript.
